# Rights Metaphors Across Hybrid Legal Languages, Such as Euro English and Legal Chinese

**DOI:** 10.1007/s11196-020-09814-6

**Published:** 2021-04-03

**Authors:** Michele Mannoni

**Affiliations:** Department of Foreign Languages and Literature, University of Verona, Verona, Italy

**Keywords:** Conceptual metaphor theory (CMT), Cognitive equivalence, Corpus linguistics and big data analysis, Chinese legal language, Rights and *quanli* 权利

## Abstract

This paper focuses on two legal languages such as the legal English developed by the European Union institutions (Euro English) and the legal Chinese of Mainland China, to study whether the mental representations and the embodied simulation created by the conceptual metaphors for the same Western concept, right, differ in any significant ways. By analysing the data contained in two large corpora, this study has found that, despite the common origin of the concept right in the two legal languages, they conceptualise it in a significantly different fashion. Finally, the findings of this study are read through the theoretical framework proposed for this special issue—hybridity and the Third Space. While it is somewhat straightforward to conceive of Euro English as a hybrid language, owing to the multilingual and supranational setting where it is used, this study has found that the Chinese legal language, too, is a hybrid language exhibiting linguistic features that intersect different belief systems.

## Introduction

Justice Benjamin Cardozo famously admonished against the use of metaphors in legal language: “Metaphors in law”, he argued, “are to be narrowly watched, for though starting as devices to liberate thought, they end often by enslaving it” [16].

About 50 years after Cardozo’s warning, Lakoff and Johnson [41] published a famous study, *Metaphors We Live By*, that radically changed the path of many fields of scholarly enquiry in semantics and other areas relating to meaning-making, including semiotics. The two scholars have shown that metaphor is not an ornamental device that we can make short of. Rather, metaphor is a fundamental tool of our cognition that we need to make sense of the reality around us. Our abstract thinking is metaphorical in nature. We cannot but resort to metaphors whenever we talk about abstract concepts, such as emotions, morality, and, notably, legal concepts. Metaphors are so much everywhere that we don’t notice them, and we use them without any effort whatsoever. When we say ‘This will cheer him up’ and ‘I’m on cloud nine’ or ‘I’m feeling down today’, we are using metaphors. Since ‘up’ and ‘cloud nine’ do not literally mean happiness, and ‘down’ does not literally mean sadness, there is a *semantic tension* [18] between the literal meanings of these words and their meanings in context that makes these words metaphorical. Following the path opened by Lakoff and Johnson, metaphor is defined in cognitive linguistics as “understanding one conceptual domain in terms of another conceptual domain” [39, 4]. In the above examples, the conceptual domains^1^ of up and down are used to talk about and conceive of happiness and sadness, respectively.

Law is packed with abstract concepts. Consequently, metaphor is ubiquitous in law. Justice, injustice, tort, damages, rights, wrong, etc. are all abstract concepts that are talked about metaphorically on a daily basis in law schools and tribunals of countries all over the globe. The very Latin word *lex/legis* from which the word *legal* evolved is itself a metaphor, for it meant ‘bundle of tied sticks’: The Ancient Romans used the metaphor to indicate that the law was binding [3, 43]. Although we no longer conceive of law as a bundle of tied sticks, we continue to speak of law as such, as we say that the law is *binding*. Even more importantly, this metaphor prompts us to *act* accordingly, as though we were physically tied up by the law. In this regard, it has been noted that metaphors of law are *jurisgenerative* (Cover 1983, in [29], i.e., they create law (e.g., [43]).

Some of the metaphors that are used to create and enforce legal concepts in court may be the same in different languages and cultures (which I regard as a continuum, drawing from Agar’s notion of *languaculture*; [2]). For instance, in English, the very word ‘binding’ has a literal meaning ‘to tie up’ that is used metaphorically in the legal context (e.g., a *binding* agreement). Similarly, in Chinese, a disyllable word *yueshu* (约束) is used to say the same, with both its components literally meaning ‘(to) tie’. Some other metaphors, though, may be more culture specific or poorly represented, when not absent, in some other languacultures. Translation may be one way that forces metaphors to be imported from one languaculture to another. In this connection, it is noted that the legal transplantation process of legal notions from one legal system to another inevitably requires translation.

Between the end of the 19th and the beginning of the 20th centuries, many legal concepts of continental Europe were imported to China. In the process, legal concepts which were new and, consequently, obscure to the Chinese entered the country through translation. The Chinese thus created a specific legal language to import these notions—a *hybrid* language, as we will see. In more recent years and in a different context, the 1992 Maastricht Treaty gave birth to the European Union (EU), which now has 24 official languages but uses English as a “legal *lingua franca*” [53, 184], which, as has been noted, is “a *hybrid* language” [53, 201]; my emphasis) in the multilingual context of the EU institutions, and one “which is not connected with a given system of values” (*ibid*.). However, one might ask *whether the mental representations of Western legal concepts as revealed by metaphors are the same in the EU and in China*. In a broader perspective, and consequently, this raises the issue of *whether hybrid languages are in fact ‘system*-*bound’* [12, 23] *or if they are ‘value*-*free’*, and, in a sense, whether there is one-Third Space or multiple Third Spaces [9, 10], each with features of its own.

This paper focuses on a plainly Western [30, 943] legal concept, right, to study whether its metaphors in two hybrid legal languages such as the legal Chinese of Mainland China^2^ (hereafter legal Chinese) and the legal English developed by the European institutions (hereafter Euro English) differ in any substantial way. First, I contextualise my study in relation to the cognitive view of metaphor and put it in a cross-lingual perspective benefitting from the intervention of translation studies. Second, I explain my data drawn from two language corpora, one for each of the language varieties under analysis, and the method of enquiry, and highlight how to compare metaphors cross-lingually. Third, I actually show and compare the metaphors for right as found in Euro English with those found in legal Chinese. Finally, I discuss the findings of this study and relate them to the foci of this special issue—hybridity and Third Space in legal translation.

## Background

In cognitive linguistics, the field of enquiry that most welcomed and further developed Lakoff and Johnson’s input, we distinguish two types of metaphors: metaphors in language and metaphors in thought. The first are the ones that we find in the language we use when writing or speaking and are commonly termed *linguistic metaphors*. In the examples I have used before (‘This will cheer him up’, etc.), ‘up’, ‘cloud nine’ and ‘down’ are linguistic wordings presenting a semantic tension between their literal and contextual meanings which makes them linguistic metaphors. The literal meaning metaphor scholars look for is more accurately termed *basic* or *primary meaning*, that is, a meaning that is more concrete, more body related, more precise and historically older than the one in context. Whenever there is such a semantic tension there is metaphoricity. The Ancient Greek term for metaphor, *metaphorá* (*μεταφορά*), comes from the word *metaphérō* (*μεταφέρω*), meaning ‘I carry’. Indeed, in metaphors there is one *source domain* (SD) which is mapped (i.e., carried, so to say) onto a *target domain* (TD). The SD is physical, concrete, and bodily related, while the TD is abstract and less physical and less bodily experienced. In the above examples, the SDs of up and down are mapped onto the TDs of happiness and sadness, respectively.

Linguistic metaphors are said to *instantiate* metaphors in thought, which are termed conceptual metaphors (hereafter CMs, or CM when used as an acronym for the singular form). Accordingly, the theoretical framework laid down by Lakoff and Johnson and developed by many metaphor scholars (e.g., [18, 20, 24, 26, 40, 62, 76] is called conceptual metaphor theory (CMT). CMs are conventionally expressed in scholarly works in the formula td is sd (in small caps). For the three examples at the outset, we can use the formulae happiness is up and sadness is down to express the CMs that the wordings realise.

A linguistic metaphor happens in the language, while a CM happens in the mind and in the body. For instance, Matlock [48] has shown that when someone is exposed to a certain metaphor, they actually simulate the meaning of the metaphor in their body (a phenomenon termed embodied simulation). By repeatedly hearing and uttering different realisations of the same CM, we reinforce its mapping. In so doing, our neuronal system is engaged, prompting us to physically experience metaphors. Indeed, metaphors are way more than mere embellishments of language. It is in this perspective that comparing metaphors between languages shall not be seen as an intellectual exercise of mere identification of the rhetorical devices that different language users use; rather, it is a comparison of what different peoples of different cultures experience in their minds and, connectedly, in their bodies.

In cross-lingual studies, the description (in Toury’s terms [66]) of metaphor variation can be contrasted with the oft-cited approach set by Newmark [52] for the translation of metaphors. However, Newmark did not focus on CMs, the study of which had just begun at the time of his 1981 work, and his approach was prescriptive rather than descriptive. Schäffner ([61], 1267) highlighted the importance of CMT for translation studies more than fifteen years ago, arguing that the study of texts with respect to CMs and metaphorical reasoning can assist the researcher in gaining better awareness of the cultural differences in conceptual structures in different languages. However, as of the time of this paper, only a handful of studies has taken a cognitive approach to the study of metaphors in specialised languages (e.g. [63], let alone legal languages. For the purpose of comparing different CMs in the two languages under analysis here, we can exploit the notion of *cognitive equivalence* used by Mohammad Q. Al-Zoubi et al. [4] in reference to the translation of CMs. They reportedly incorporated Katan [37, (27)]’s view, according to whom, *cognitive equivalenc*e is the equivalence between two phrasings, not in terms of their literal meanings, but in terms of the ‘forms of things’ that the respective users have in mind as revealed by the metaphorical mappings that the phrasings instantiate. The same principle has been termed by Mandelblit [44] *similar mapping condition*, i.e. the condition by which two linguistic metaphors instantiate the same CM, irrespective of the differences between the linguistic utterances. In the following section, I will show how I proceeded to compare the mappings of right in the two languages, i.e. Euro English and Chinese, in order to ascertain whether there was cognitive equivalence between them.

When it comes to other forms of equivalence between Chinese legal terms and the terms of other languacultures, right has particularly been the focus of the scholars’ attention. Various academics have shown that despite a surface translational equivalence between the English word ‘right’ and the Chinese correspondents *quanli* 权利 (in some cases abbreviated as *quan* 权), notable differences in their legal meanings can be detected [13, 14, 45]. This may be attributable to the fact that, as said, the Chinese legal system is not native of China, and the modern legal concepts that we find in it have mostly been transplanted from the West. Indeed, “legal concepts are the results of the ‘stratification’ of different meanings which have been developed by the various traditions—even within a particular legal system—over the course of time” [53, 186]. As we will see in the analysis, the mental space created by the transplantation and translation of right in China is a hybrid one—a Third Space, in Bhabha’s acceptation [10].

Before turning to the analysis, I will show the method and the linguistic data I used.

## Method of Analysis and Data

### Corpus Search

One may well wonder where we *materially* find linguistic metaphors. In fact, the answer is simple and has already been given above: they are found in language. But *where* exactly in language, when one has thousands or millions of words to analyse? The answer becomes more complicated.

There is no established procedure that helps us to promptly identify linguistic metaphors in big datasets, such as large language corpora. Indeed, some metaphorical meaning may be retained in the etymology of a word, as is the case with the Latin word for ‘legal’, *lex/legis*, as we saw above. More importantly, the etymological meanings of a word may have left traces in the modern variety of a language, as is the case with an agreement which, when it is legal is said to be binding for the parties to it, but no concrete laces tie them up.

Various prominent metaphor scholars suggest that we perform a word-by-word analysis of an entire text or a corpus of text to look for semantic tension in each and every lexical unit (see e.g., the MIP and MIPVU procedures by Pragglejaz Group and Steen et al. [54, 64], respectively). Whenever a lexical unit exhibits such a tension, we can mark it as metaphorical. It is noted that both MIP and MIPVU instruct the researcher to look up a word’s more basic and primary meanings, rather than its contextual meaning in a dictionary, and such is the procedure I adopted. This is feasible when one has a text or a language corpus whose dimension is actually—not just virtually—manageable by an individual researcher or a research team. Additionally, procedures such as MIP and MIPVU do not suggest how to proceed in case of a keyword-based analysis, as is our case here. If we are to look for the linguistic metaphors that ‘right(s)’ or ‘*quanli*’ realise, as I did for this study, we may not be interested in analysing each and every word of a text where, one may sadly end up finding, my keyword is scarcely used, if ever. Moreover, many corpus managers, such as Sketch Engine [38], do not grant the researcher full access to the texts. All these aspects may complicate the searching for metaphors in texts.

One method that is commonly adopted to swiftly analyse large datasets (see e.g., Semino [62] is to prompt the software to show us the words (*collocates*) that are strongly bound to a keyword (*node*). This is the method I used for the research I present here. There are various statistical measures implemented in corpus software facilities that find collocates, each of them highlighting some aspects of the relation with the node and hiding others (e.g., [25, 161–62]. I decided to use logDice, given that it “has a reasonable interpretation, scales well on a different corpus size, is stable on subcorpora, and the values are in reasonable [pre-set] range” [60, 7]. Its maximum value is 14, when two words always and symmetrically attract each other (e.g., *zig zag*). General values are below 10 [60, 9]. For instance, if we were interested in the metaphors that ‘up’ realises, by using logDice we may find ‘climb’ among the collocates, if the linguistic expression ‘climb up’ is recurrently used. Moreover, since logDice works on a pre-set scale of 14, it enables comparability across corpora of different sizes. I searched the collocates of my nodes in a ten-word span window, i.e. five words right and five left of the nodes. In order to evaluate if the contextual meaning of a collocate contrasted with a more primary and basic meaning, I concordanced^3^ each collocate. For the purposes of this preliminary study, a sample of data consisting of the first hundred strongest collocates were considered.

I can now illustrate the two corpora I used for the languages under analysis and the linguistic data they contain:

- For Euro English I used the EUR-Lex English 2/2016 corpus [7], available through the online corpus manager SketchEngine mentioned earlier, access to which was provided to me by my university. The corpus contains 193.3 million words. It is, thus, a large corpus. It contains various types of legal documents, including EU law (EU treaties, directives, regulations, decisions, consolidated legislation, etc.), EU caselaw, preparatory acts, etc.^4^ Thus, it is believed that this corpus is representative of the legal language variety used at the EU institutions. In order to search for the collocates of ‘right’ and ‘rights’ at once, I used the CQL (Corpus Query Language) formula [word = “right|rights”].
- For legal Chinese, I used a corpus of Chinese laws (ChinLaC, a provisional acronym) that is being built at my department at the University of Verona, Italy, under the Project of Excellence plan in ‘Digital Humanities Applied to Foreign Languages and Literature’. The corpus, soon to be freely accessible online, counts 1.5 million words so far and includes the laws and regulations in force up to December 2019 except for some criminal laws whose integration into the corpus is still ongoing. The software facility I used for its consultation is #LancsBox 5.1.2 [11].

The reference dictionaries I used to check if there was any semantic tension between a basic and a contextual meaning are *Macmillan Dictionary* for English, and *Xiandai Hanyu Cidian* (XHC) for Chinese. For the purpose of finding the etymological meanings of the words under analysis, I used Harper’s *Online Etymology Dictionary* for English and *Le Grand Ricci Numèrique* [6] for Chinese.

### Metaphor Decomposition

One final aspect that needs to be illustrated before coming to the results of my metaphor search is what types of metaphors are most suitable for cross-lingual comparison. It is proposed that when we compare metaphors for the same concept in different languages with an eye to ascertaining whether the mental representations for it exhibit significant differences, we need to compare the most basic and most body-related levels of mappings.

In this respect, we can identify two types of metaphors, following inter alia Grady [31, 32]: *primary metaphors*, the ones I actually compared, and *complex metaphors*. With regard to Grady’s theory, Lakoff and Johnson offered the following definition:

All complex metaphors are “molecular,” made up of “atomic” metaphorical parts called *primary metaphors*. Each primary metaphor has a minimal structure and arises naturally, automatically, and unconsciously through everyday experience by means of conflation, during which cross-domain associations are formed. *Complex metaphors* are formed by conceptual blending. Universal early experiences lead to universal conflations, which then develop into universal (or widespread) conventional conceptual metaphors.

[42, 46]

Thus, for instance, ‘Climbing the corporate ladder’ is a linguistic metaphor realising gaining power is moving up, a complex metaphor drawing from a purposeful life is a journey, a simpler one, and encapsulating power is up. Similarly, in Chinese, *ta you le hen da de jinbu* (他有了很大的进步 ‘He has made much progress’, with *jinbu* ‘progress’ literally meaning ‘step forward’) realises progress in life is distance covered along a path (cf. [27, 25]). This metaphor, too, draws from a purposeful life is a journey. Therefore, although ‘Climbing the corporate ladder’ and the Chinese sentence convey different meanings with different aims in different contexts, they indicate that both Chinese and English speakers conceive of life as a journey, i.e. life is a journey. Additionally, the two examples draw from a primary metaphor such as purposes are destinations. This primary metaphor is very bodily related: when we want to get an object, say, a bottle of water, we have to physically move towards it, thus conceiving of the object as both the *purpose* and our *destination*. Were we focussing our research on life, we would have found a similar conception of it in two languacultures, such as the English and the Chinese. In this study, I adopted such approach to the study of right metaphors.^5^

Besides identifying primary metaphors, one further way to get even more primary, so to say, is to identify the patterns grounded in our body from which primary metaphors themselves originate. In CMT, these are called *embodied schemas* (or ‘image schemas’, or simply ‘schemas’), which have been defined as

a recurrent pattern, shape, and regularity in, or of, […] ongoing ordering activities. These patterns emerge as meaningful structures for us chiefly at the level of our bodily movements through space, our manipulation of objects, and our perceptual interactions.

[36, 29]

Consequently, since schemas directly emerge from the interaction of our bodies with the environment around us, they are extremely skeletal, very few in number and preconceptual. Evans and Green [22, 190] elaborated a partial list of image schemas (e.g., space: up-down; front-back, left-right, etc.; balance: twin-pan balance, etc.; existence: bounded space; object; etc.; containment: container; in-out; etc.), drawing on the influential research of Cienki (1998), Gibbs and Colston (1995), Johnson [36], Lakoff (1987) and Lakoff and Turner (1989) (all cited in *ibid.*).

For instance, in ‘Climbing the corporate ladder’ that we have just seen, the primary metaphor purposes are destinations is instantiated by the source-path-goal embodied schema, while power is up by up-down and verticality.

It is believed that by comparing not just metaphors and primary metaphors, but also image schemas between different languages, we can obtain a more accurate and body-related picture of how metaphors in language are grounded in the body of the respective speakers. Such is the approach I took for the study of right metaphors in Euro English and Chinese.

## Analysis

I now move to illustrating and discussing the metaphorical collocates of the nodes ‘right(s)’ and *quanli* that I found in the two languages under analysis, Euro English and legal Chinese. In the analysis I also considered the etymological meanings of the nodes, so to see if they have left traces in the contemporary varieties of the language. I start with Euro English rights metaphors.

### Rights Metaphors in Euro English

Table 1 resumes the metaphors and the image schemas I found by analysing the etymology of the word ‘right’ and its metaphorical collocates in Euro English, which I now turn to discussing.

**Table 1:** Metaphors for the words ‘right’ and ‘rights’ in Euro English as found in its etymology and in the sampled data from the corpus EUR-Lex English 2/2016

‘Right’ etymology		Conceptual metaphor	Embodied schema
*riht* *reg- *orektos* *rectus* *…*		good is right (i.e. straight, not-bent)	straight
		good is right (i.e. upright)	verticality
		the best road is the straightest one	source-path-goal
		good is right (i.e. not left)	left–right
**‘right(s)’ collocate**	**logDice**		
exercise	10.06	a right is a part of the human body	ø
exercising	7.89		
exercised	7.57		
affect	7.76		
protection	9.33	protecting rights is war	bounded space
protect	7.66		
defence	9.71		
violations	8.00	protecting/violating/defending rights is protecting/violating/defending a bounded space	
have	8.93	a right is an object	object
use	8.08		
holder	7.79		
enjoy	7.76		
conferred	8.54		
acquired	8.48		
transfer	7.65		
reserves	7.64	rights are resources	
under	8.42	rights are under	up-down

First, I discuss the etymological meaning of the word ‘right’. In Harper’s etymological dictionary, we find the following explanation for the etymology of the word when it means ‘morally correct’:

Old English *riht* “just, good, fair; proper, fitting; *straight, not bent, direct, erect*,” […] from […] [Proto-Indo European] root *reg- “move *in a straight line*,” also “to rule, to lead *straight*, to put *right*” (source also of Greek *orektos* “*stretched out*, *upright*;” Latin *rectus* “*straight*, *right*;” Old Persian *rasta*- “*straight; right*,” *aršta*- “rectitude;” Old Irish *recht* “*law*;” Welsh *rhaith*, Breton *reiz* “just, righteous, wise”).

(‘right’ – *Online Etymology Dictionary*; my emphasis)

As can be seen, the current moral and legal meanings attached to the word ‘right’, meaning ‘just, good, fair’ *vel sim*, come from the physical, concrete and body-related meanings straight, i.e. not bent, and upright. The etymological reconstruction that Harper indicates also shows that such meanings were maintained in various distant languages, including Old Persian, Old Irish, and Latin. In Old Irish the word also means ‘law’, suggesting that upright had a positive moral connotation, which was then transferred onto the law domain. The etymological reconstruction suggests the existence of the metaphors good is right (i.e., straight, not-bent) and good is right (i.e., upright) not just in ancient languages, but in contemporary English as well (e.g., [39, 23–24].

As to metaphor decomposition, it is noted that straight and upright are embodied via two embodied schemas, straight and verticality, respectively. The presence of these two schemas in moral metaphors have been observed in various languages, including Chinese and English [78, 79]. There may be various reasons for the success of these schemas in morality, including the very bodily-grounded fact that when our health conditions are good we have an upright posture and our spine is straighter than when we feel sad or in pain and we feel the urge to lie down (i.e. *not upright*) and double over in pain (i.e., *not straight*). Moreover, the fact that a *straight* line between two points is shorter than a curved one may also instantiate the source-path-goal embodied schema: when we aim at something that is far from us, getting *right* to it demands less movement, less energy and less time than if we reach it through any other path (e.g., [79, 111]). The time I am referring to here is not a commodified one—that would not be directly embodied. I am speaking of the time one needs to grab something or to hide from an enemy or a predator, the sense of which may be genetically transmitted to us by our ancestors, and be more primary and primitive, and thus also more embodied.

Another etymological aspect that we can consider is the one that connects the word ‘right’ to its other meaning ‘opposite of left’. According to Harper, this latter meaning comes from

*riht*, from Old English *riht*, which did not have this sense but meant “good, proper, fitting, straight” […]. The notion is of the right hand as the “correct” hand.

(‘right’– *Online Etymology Dictionary*; my emphasis)

According to this reconstruction, the sense of right as opposite of left is less concrete and less physical than may seem at first, given that ‘the right hand’ in fact means ‘the *correct* hand’, with ‘correct’ being more abstract than ‘opposite of left’. In this regard, the underlying metaphor is good is right (i.e. opposite of left). The embodiment of this metaphor has been proved by experiments [17] showing that right-handed people tend to conceive of the space on their right as good and that on their left as bad. Since the vast majority of people are right-handed, right is generally conceived of as good (*ibid*., 353).

To recap, based on the etymology analysis that I have just illustrated and some insight from contemporary metaphor research, it seems that English users conceive of straight, upright, and not-left as good. For our purposes, it is suggested that these metaphors profile right as being inherently good. To put this in more legal terms, rights are lawful and just entitlements by definition, so that one cannot speak of, say, ‘illegal rights’ in English.

We can now turn to the analysis of the metaphors that ‘right(s)’ as a just entitlement instantiates when used in the Euro English legal language as represented in my data. The strongest collocate that I could mark as metaphorical is ‘exercise’. Indeed, the *Macmillan Dictionary* indicates that the word ‘exercise’ has a primary meaning that contrasts with the one in the legal context as shown by its concordances in the corpus:

to move or use a particular *part of your body* in order to make it strong. [E.g.] The doctor said I should *exercise my* knee every morning.

(‘Right’ – *Macmillan Dictionary*; my emphasis)

This primary meaning is concrete and body-related, and is less abstract than the metaphorical meaning ‘to use’ that the verb has in the sampled sentences, such as (1), (2) and (3):

1. More favourable conditions should therefore be laid down for the *exercise* of *their right* to family reunification.
2. Therefore, it must be held that the Court’s interpretation of Article 2(1) of Regulation No 1408/71 does not, as such, have any impact on the worker’s choice as to whether or not to *exercise his right* to freedom of movement.
3. In the aforesaid situations at least there is no autonomous abusive conduct which is independent of the *exercise of the* intellectual property *right* in question as was the case in the first two examples in […]
  (EUR-Lex English 2/2016; my emphasis)

Thus, one can physically exercise any particular part of their bodies to make them stronger and more powerful, and one can metaphorically do the same with their rights in order to make them stronger in court. This interpretation is further confirmed by the presence of the possessive adjectives in (1) and (2) (‘exercise of *their* right’, ‘exercise *his* right’), as well as in the example provided by my reference dictionary, in which the possessive adjective is used in a similar fashion. Importantly, this suggests that rights in Euro English may be strongly embodied, as they are conceived of as a part of the human body. This instantiates the metaphor a right is a part of the human body. This is a strong metaphor that is reinforced by another linguistic metaphor ‘affect’ (logDice 7.79) that the search retrieves. The word ‘[to] affect’ has the primary meaning of ‘to cause physical damage to something’, including our body and our body parts (‘Affect’—*Macmillan Dictionary*)

(4) The disease affects many different *organs of the body*.
  (‘Affect’ – *Macmillan Dictionary* – my emphasis)

This more concrete and body-related sense of the verb ‘to affect’ contrasts with the less physical and more abstract and vaguer one that the word has when it occurs with the node ‘right’, as in the following sentence sampled from the corpus:

(5) Termination or expiry of the Agreement shall not *affect rights* or obligations in accordance with this Annex.
  (EUR-Lex English 2/2016; my emphasis)

There is a semantic tension between ‘affect a body part’ in (4) and ‘affect rights’ in (5) that reinforces the metaphor a right is a part of the human body. The possible diffusion of this CM in many Western languacultures may provide an additional explanation as to why rights are so salient in contemporary Western cultures, and why people strive for them and feel so violated when their rights are affected. This may also suggest that in the Western and, specifically and for our purposes, EU conception, rights are grounded in the body—a thesis maintained inter alia by Mooney [51] with specific reference to human rights more generally.

The following three strongest collocates that my search found activate the war domain. These are ‘defence’ (logDice 9.71), ‘protection’ (logDice 9.33) and ‘violations’ (logDice 8.00). The first of these, ‘defence’, is defined as the

actions that you take to protect someone or something that is being attacked […] [and] the system of weapons, equipment, and people that is used to protect a country

(‘Defence’ – *Macmillan Dictionary*)

As can be seen, the war domain is especially instantiated by ‘defence’ when rights are endangered. The word ‘defence’ activates defence, a concept which underlies the legal term ‘defendant’, suggesting that the entire court process as described in English may be conceived of in terms of war (see also [58, 6–7]). Western speakers are used to thinking of the court trial as a fight and the court as a battlefield, where one party *wins*, the other *loses*, they both need a *strategy* to argue their case. It is in this battlefield that rights are defended.

The third strongest collocate of ‘right(s) is ‘protection’ (logDice 9.75). According to my reference dictionary, it means

the process of keeping someone or something *safe*, or the condition of being kept *safe*

(‘Protection’ – *Macmillan Dictionary*; my emphasis)

This meaning is similarly attributed to the verb ‘to protect’, also appearing among the collocates of ‘right(s)’ (logDice 7.69). The corpus finds various occurrences of the word ‘protect(ion)’ in which the word ‘protect’ is used in connection to ‘rights’ in an abstract and non-body related sense:

(6) The Court ensured *the protection of* fundamental *rights* and freedoms whilst recognising the imperative need to combat international terrorism.
  (EUR-Lex English 2/2016; my emphasis)

In (6), right is either personified or objectified and the Court decided to protect fundamental rights and freedoms while allowing the combatting of international terrorism. It has been proposed that when the war domain is instantiated, drastic measures, including violent ones, such as war, may be more easily justified (e.g., [39, 68–69]), although this is not necessarily always the case (e.g., [23]). Metaphorically speaking, and for our purposes, defending right implies a struggle, i.e., defending right is war.

Also, it should be noted that in war, space plays a key role. A battle is won when the adversary troops advance and invade the space of their enemy. At the end of a war, as we *intersubjectively*^6^ imagine it, a space ends up being occupied by the adversary. It is suggested that the space domain and, more precisely, the bounded space schema, is activated by war and especially by a collocate of ‘right(s)’, ‘violations’ (logDice 8.04), basically meaning

the action of entering an area or place without permission

(‘Violation’ – *Macmillan Dictionary*)

Indeed, the peoples of some cultures may instinctively *protect* and *defend* the space that they consider to be theirs from *violations*. In other words, this may instantiate the metaphors protecting/violating/defending rights is protecting/violating/defending a bounded space. This seems to suggest that in the EU conception, rights are strongly conceived of in relation to private property—a vision that one may expect to emerge from the English language of an Anglo-centred legal culture, in which rights are firstly and foremostly the rights to private property, following Locke and Hobbes (see e.g., [72], 403–4). This metaphor is consistent with one of the right metaphors, rights are territories listed in MetaNet, the repository of conceptual metaphors housed at the International Computer Science Institute in Berkeley, California ([50]). As has been noted, metaphor is not culture free; quite the reverse, it is imbued with ideology (e.g. [28]). This has implications for the definition of Euro English as a hybrid language, as it contrasts with the idea that this language is not connected to a given system of value.

We can now turn to examining the other collocates of ‘right(s)’. Legal rights are abstract in nature. That is, in essence, why they are talked about metaphorically. It would be impossible for us to conceive of them as totally immaterial substances. The data reveals that in Euro English they are treated as if they were very material and tangible entities (a right is an object). The metaphors serving this purpose, *ontological metaphors* [41, 25, 39, 38–39], make it easier for us to understand and operate with abstract entities, however scarce the knowledge that we have of the SD, object, is. For instance, the data shows that people ‘have’ (logDice 8.91) them and ‘use’ (logDice 8.07) them, and when one is entitled to them, they are said to be ‘holder[s]’ (logDice 7.51) of them and can ‘enjoy’ (logDice 7.79) them. People can also store them to ensure that there is no shortage of them; in fact, the corpus reveals that one ‘reserves’ (logDice 7.68) them for future need (hence, rights are resources). All these words have primary meanings that are related to concrete—rather than abstract—objects. For instance, when one holds something, they ‘carry *something* using [their] *hands* or *arms’* (‘Hold’—*Macmillan Dictionary*; my emphasis). Likewise, ‘to enjoy’ means ‘to get pleasure from *something’* (‘Enjoy’—*Macmillan Dictionary*; my emphasis); ‘to reserve’ means ‘to keep *something* so that you can use it when you need to’ (‘Reserve’—*Macmillan Dictionary*), and so on. These meanings contrast with the contextual meanings that these words have in the legal discourse of the EU:

(7) Citizens of the Union shall *enjoy* the *rights* and be subject to the duties provided for in the Treaties.
(8) It therefore *reserves* the *right* to come back to this aspect during the second reading.
  (EUR-Lex English 2/2016; my emphasis)

Clearly, the citizens in (7) are not getting pleasure from rights, nor is the subject of (8) physically setting aside them. All these metaphors use the object embodied schema. This schema “is based on our everyday interaction with concrete objects […] [and] generalises over what is common to objects: for example, that they have physical attributes such as colour, *weight* and *shape*, […]” [22, 191]; my emphasis). It is through this schema that we can also appreciate other collocates that the corpus search retrieved, such as ‘conferred’ (logDice 8.54), ‘acquired’ (logDice 8.48), ‘transfer’ (logDice 7.65). In fact, just like if ‘rights’ were concrete objects, they are moved through space from a giver to a receiver, and talked about accordingly.

The last metaphorical collocate of ‘right(s)’ to be discussed is the word ‘under’ (logDice 8.42). The physical primary meaning that the dictionary indicates for this word is ‘directly below or at a lower level than something’ (‘Under’—*Macmillan Dictionary*). In contrast, the word is used in the corpus in sentences such as the following, where it metaphorically means ‘according to a particular law, agreement or system’ (*ibid*.):

(9) Access *right under Regulation* (EC) No 1986/2006
(10) Furthermore, having acquired no *rights under* the old *legislation*, the applicant could not claim to be entitled to any transitional measures for his benefit.
(11) the number of *rights* granted *under Article* 118(3) of Regulation (EC) No 1782/2003 during the preceding year;

As can be noticed by contrasting the instances found in the corpus and the primary meaning of ‘under’, there is sufficient semantic tension to mark the word as metaphorical. In fact, a right cannot be *physically* and *materially under* something else, nor does the primary meaning suggest that the phrasing means compliance with a law or a legal provision. It seems that the CM that emerges from the examples is a right that is created by a law is under it, a complex metaphor that may be decomposed as follows:

- a right that is created by a law is under it
- a right that is created by a law complies with it
- complying with a law is being under it
- obeying something or somebody is being under it
- control is up/lack of control is down

The final primary metaphor that we found, an orientational metaphor, is widespread in many languacultures, if not universal [39, 40]. It is embodied through the schema up-down.

I will now turn to examining the metaphorical instantiations of right in legal Chinese, and compare them with those of Euro English that we have just seen.

### Rights Metaphors in Chinese

As I have done for right in Euro English, I first analyse the etymology of the Chinese word for ‘right(s)’, *quanli*, and then move to discussing its metaphorical collocates in my data. In Table 2 above I show the results of my metaphor search for the Chinese legal term *quanli*.

**Table 2:** Metaphors for the word quanli (权利 ‘right(s)’) in legal Chinese as found in its etymology and in the corpus ChinLac

*quanli* (权利) etymology		Conceptual metaphor	Embodied schema
*quanli*		ø	ø
*quan*-		weighing is evaluating	balance
-*li*		ø	ø
**‘right(s)’ collocate**	**logDice**		
*baohu* (保护) ‘protect/protection’	9.17	protecting a right is protecting an object from being damaged	object
*qinhai* (侵害) ‘to invade-harm’	8.63	rights are barriers protecting a space + damaging a right is damaging an object	bounded space + object
*you* (有) ‘to have’	8.94	rights are objects	object

First, as to the etymological meaning of the word, it is not possible to find it in Classical Chinese. Indeed, although *quan* and *li* occurred together on some occasions, the two did not form an individual word. W. A. P. Martin (1827–2910) of the American Presbyterian Mission is credited with inventing the word *quanli* to translate the Western notion of rights in his Chinese translation [71] of Wheaton’s *Elements of International Law* (e.g., [65, 131]). Notably, *quanli* is not a Chinese word in the sense that it was not invented by the Chinese and, as has been noted, the very concept of right has no traces in traditional Chinese philosophy and culture, including legal culture ([13]; but cf. [35]^7^). Accordingly, right can be regarded as a Western concept that has been introduced in China only recently, along with many other legal concepts which entered the country between the end of the 19th and the beginning of the 20th centuries. Consequently and for our purposes, we must conclude that there could be *no* semantic tension between a primary and a contextual meaning of the word in Classical Chinese, and today there can be no metaphorical mapping realised by the etymological meanings of the word.

However, we may want to push this boundary and separately study the old meanings of the word components, *quan*- and -*li*, to see if they did instantiate any metaphorical mappings in the past that may potentially be retained in present days. As to *quan*-, *Le Grand Ricci numèrique* indicates that it primarily meant ‘calibration weights’ (*poids de balance*) and, metonymically, ‘to weigh, to examine’ (*peser, examiner*). Its other meanings of ‘evaluate’ and ‘authority’ are metaphorical and are derived from the primary ones, given that the person who metaphorically *weighed* abstract notions (e.g., the pros and cons of situations) was in fact *evaluating* them and had the *authority* and *power* to do so (with all these meanings still attached to the word *quan* and many of its components in modern Chinese). In other words, there was a CM weighing is evaluating that was active in Classical Chinese. This suggests the existence of a complex metaphor,

- weighing is evaluating
- situations are objects (to be weighed)
- the one who weighs objects has the authority (to do so) (cultural belief)
- calibration weights stand for the scale which stands for the action of weighing (metonymies)

This is very different from what we have seen for the etymological meanings of the word ‘right’ in Euro English. The metaphorical mappings are completely different, and the straight, verticality, source-path-goal and left–right schemas that ‘right’ in English activates are not activated by the etymological meanings of *quan*-.

As to -*li*, the second component of the word *quanli*, since its first appearances on oracle bones (ca. 2nd millennium BCE) it was used to mean ‘advantage[ous], profit[able]’, mostly in a negative sense (see e.g. [65]. Its use was not metaphorical, given that there was no semantic tension between its meanings. The primary meaning of ‘harvesting grain’ that can be appreciated by observing the components making the character for -*li* (利, made by 禾 ‘grain’ + 刂 ‘knife’ = ‘cutting grain’ and receiving *profit* from it) is long lost and has no trace in modern or pre-modern Chinese.

To recap the etymological analysis, *quanli* did not instantiate any metaphor, although *quan*- individually did, whilst -*li* did not. Moreover, the component *li*- in *quanli* used to have a negative connotation. For now, suffice it to note that, contrarily to what we have seen for the etymology of right, chinese right is not inherently good. Indeed, in a separate study undertaken by the author, partly carried out in collaboration with Prof. Deborah Cao, we have found that Chinese users may speak of ‘illegal rights’ (*feifa quanyi* 非法权益) or ‘improper rights’ (*bu zhengdang quanyi* 不正当权益) with reference to rights that are not just and the law shall not protect (see e.g. (15, 46, 47)—an oxymoron in Western languages. When the court finds that a party is seeking the protection of an illegal right, the Chinese judge will normally reject the party’s claim. Considering the above, it seems that rights in the Chinese languaculture are not inherently *just* entitlements as they may be in other languacultures, such as the Euro English one. This is true in terms of legal and metaphorical meanings as well.

Second, as to the search for metaphorical collocates of *quanli*, the strongest one is *baohu* (保护 ‘protect’), with a logDice of 9.17.^8^ The word has a primary meaning ‘try one’s best to take care and prevent from being *damaged*.’ (‘*Baohu*’—XHC; my own translation and emphasis). A search for the collocates of *baohu* in a separate large corpus (of 13.5 billion words) of non-specialised Chinese, zhTenTen17 [80], similarly available through SketchEngine, shows that *baohu* is used with various tangible objects (e.g., *wenwu* ‘relics’, *senlin* ‘forests’, *shengwu* ‘organisms’) and objectified entities (e.g., *huanjing* ‘environment’, *maoyi* ‘commerce’, *xinxi* ‘information’). A primary meaning of *baohu* is then ‘protecting an object’, which is more concrete and less vague than the abstract meaning the word has when it occurs with the node, as in

(12) …保护未成年子女的权利…
  … *protect the rights* of underage children …
  (ChinLaC)

It is suggested that the use of *baohu* in Chinese law with reference to right instantiates the metaphor protecting a right is protecting an object from being damaged. Although one can say ‘*baohu yanjing’* (‘protect one’s eyes’), there is no other linguistic indication in the data confirming that *quanli* is conceived of as a part of the human body. For instance, the Chinese translation for ‘to exercise’ (*xingshi* 行使), as in ‘to exercise one’s rights’, does appear among the collocates of *quanli*, being the third strongest collocate (logDice 11.06), but has nothing to do with its Euro English correspondent that we have discussed above. *Xingshi* is a legal term that is mostly used in the Chinese legal jargon and cannot be used in relation to the human body. In fact, in Chinese one can say *duanlian shenti* (锻炼身体) ‘to exercise one’s body, to work out’, but cannot say *duanlian quanli. This contrasts with the English usage of ‘exercise one’s rights’ that we have seen earlier. In this regard, the mapping and the embodied schema found for right in Euro English language is different to the one we find in Chinese.

Even the Chinese translation for ‘to affect other’s rights’ *qinhai* (侵害) appears among the collocates of *quanli* (logDice 8.63), but is poorly connected to the human body, contrary to what we have seen for the English word it translates. *Qinhai* is a compound word made of *qin*- and -*hai*, literally meaning ‘invade’ and ‘harm’, respectively (‘*Qinhai’*—XHC; my own translation). The basic meaning is more concrete than when it appears in instances that the corpus search retrieves, such as ‘rights have been affected [i.e. infringed]’ (*quanli bei qinhai* 权利被侵害), so there might be sufficient semantic tension to mark *qinhai* in the legal context as metaphorical. Indeed, the XHC exemplifies that *qinhai* can be used in relation to the harmful action of small concrete entities, such as bacteria (e.g., ‘Prevent harmful insects from invading-harming (*qinhai*)’; XHC; my translation). Additionally, this word is used to describe what happens to our body when invisible substances and pathogens harm our health. Although it is true that, say, infections are body-related, we need to use metaphors to talk about them, since the infective process is invisible to the naked eye, and so are its causes. Thus, it is quite common in many languages to resort to war^9^ metaphors and speak of ‘viruses *attacking* cells’, ‘*defences* against pathogens’, etc. This usage of *qinhai* is confirmed in zhTenTen17 in instances such as

(13) 长期劳累,使身体防御力下降,易受*病毒侵害*引发各种疾病。
  Protracted stress reduces the defences of the body and makes it easier for it to be *invaded*-*harmed* (*qinhai*) by bacteria that can cause various diseases.

Thus, as can be seen, the verb *qinhai* implies that similarly to human bodies, rights are barriers protecting a space that can be invaded (*qin*-) and the object contained therein damaged (-*hai*), so that damaging a right is damaging an object, although the corpus shows no other linguistic metaphor that reinforces this mapping. Both the bounded space and object schemas are activated. However, a search in the non-specialised Chinese corpus zhTenTen17 shows that *qinhai* is mostly used with legal terms, as its strongest collocates include ‘rights and interests’ (*quanyi*), ‘unlawfully’ (*bufa*), ‘right to good name and reputation’ (*mingyuquan*), ‘right to privacy’ (*yinsiquan*). It is suggested that although *qinhai* may be regarded as exhibiting metaphoricity, in fact its metaphorical meaning is rather loose.

As to the other metaphorical collocates of *quanli* that I found in my data, one of them, *you* (有 ‘to have’; logDice 8.94), shows that just like in Euro English, Chinese rights are conceived of as objects, and, thus, one *have* them. Another collocate ‘transfer’ *zhuanrang* (转让; logDice 8.88) appears among the collocates of *quanli* and can be virtually regarded as being metaphorical, due to the basic meanings of its components *zhuan*- and *rang*-, meaning ‘to turn’ and ‘to give way’, respectively. However, similarly to what we have seen for *qinhai* but even more remarkably so, *zhuanrang* is a legal word and its usage is confined to the legal jargon, as a separate search on zhTenTen17 shows. For this reason, upon evaluating the use of *zhuanrang* as an individual word in non-specialised Chinese, I did not consider the word as exhibiting sufficient metaphoricity, despite the meanings that its morphemes can have in other contexts.

Finally, the search retrieved a collocate of *quanli*, *yifa* (依法; logDice 8.87), that may be used to translate ‘under’ (as in ‘under the law’), one of the collocates of ‘right(s)’ in Euro English, for it similarly means ‘in compliance with the law’. However, again, the word is a compound legal word made of *yi* (依 ‘to be closely next to’) and *fa* (法 ‘law’), and thus instantiates no metaphorical mapping, given that it cannot be used outside of the legal language, for, as can be seen, the very word ‘law’ *fa* is encapsulated in it. The concordances show that the word occurs in instances such as

(14) 依法享受诉讼的权利 […].
  Enjoy the right to stand in court *in compliance with the law* (*yifa*) […]
  (ChinLaC)

It may be worth noting that since *yi* means ‘to be closely next to’, lawful rights are conceived of as being *close* to a certain law, rather than *under* it as we have seen to be true for rights in English. The Chinese wording may suggest the existence of a widespread metaphor similarity is proximity (e.g., [42, 58]) although this CM, too, emerges only if we consider the morphemes of which *yifa* is composed, rather than from a primary meaning of *yifa* itself in contrast with its legal usage. Consequently, similarly to what I have proposed for *zhuanrang*, I did not consider *yifa* as metaphorical.

Finally, it should be noted that the translational equivalents of the English metaphorical collocates of ‘right(s)’ mostly appear among the Chinese collocates of *quanli* that my search found, although not all of them could be marked as metaphorical (as was the case with *xingshi* ‘exercise’, discussed above). As to the English words whose translation does not appear among the Chinese collocates that I considered, two of them (i.e. *shiyong* 使用 for ‘use’ and *shouyu* 授予 for ‘confer’) do not appear within my sample, while two others (i.e. *chiyou* 持有者 for ‘holder’ and *baoliu* 保留 for ‘reserve’) do not appear in ChinLaC at all. However, even if we had considered these words, the metaphorical mappings that my search revealed would not be different, since these words realise right is an object, except for *chiyouzhe* which may not realise any metaphor, being a neologism. This seems to suggest that the data sampling was correct in terms of representativeness of the unsampled population of data.

## Findings and Final Remarks

We can now discuss the findings of this study in relation to the two research questions with which we started.

As to the first question, *whether the mental representations of* *right* *as revealed by metaphors are the same in the EU institutions and in Mainland China*, by comparing Table 1 with Table 2 in light of the above discussion, it seems that the mental representations created in the two languages differ in a number of ways—both in quantitative and qualitative terms. First, from the quantitative perspective, the number of linguistic and conceptual metaphors that my corpus search found for right are higher in Euro English than in legal Chinese. Indeed, Euro English uses seventeen linguistic metaphors to realise six conceptual metaphors, while legal Chinese uses three linguistic metaphors to realise half (i.e. three) of the conceptual metaphors instantiated in Euro English. If we consider the etymological meanings of the words under analysis, metaphoricity is even higher in Euro English than in legal Chinese, given that ‘right(s)’ in English realises more conceptual metaphors and uses more schemas than *quanli* does in legal Chinese. In Euro English as represented in my data, right metaphors draw from seven different embodied schemas, while in legal Chinese right is conceptualised by less than a half (i.e. three) of the schemas used in Euro English for the same concept.

In qualitative terms, this may suggest that Euro English provides much richer a picture of what right is and how it functions in the legal context, while right in legal Chinese is more poorly delineated and potentially more obscure to Chinese users. This was especially true when the notion entered China at the end of 19th century, and the Chinese made various attempts at understanding and translating the concept right in their language, as the first attempts at translation show (e.g., ‘in case of’ *dang … zhi li* 当…之例, ‘wishing to’ *yu* 欲; [65, 129]. It was at that time that many legal neologisms were created, and these neologisms are not metaphorical, as we saw. Additionally, the etymological metaphorical meanings of the word ‘right’ account for the fact that right in Euro English and, more generally, in the West are *just* (i.e., good) entitlements by definition, whilst this is not true in Chinese, where a phrase *illegal rights* appears to be used.

Owing to the jurisgenerative power of metaphor and the embodied simulation it entails, there is scarce cognitive equivalence between right in Euro English and right in legal Chinese, and, possibly, scarce legal equivalence.

In terms of the limitations of this study, it is acknowledged that metaphoricity may be higher in the selected corpus of Euro English than in the one for legal Chinese, owing to the different genres to which the texts they contain belong, although, broadly speaking, both languages can be labelled as ‘legal languages’. One other limitation may be due to the cut-off line set at the first hundred collocates, which have captured only the strongest linguistic metaphors. However, the fact that this sampling criterion was applied to both populations of data for the two languages make it possible to assume that the phenomena observed are not skewed to any of the two languages in particular. It should also be acknowledged that, as we have seen, linguistic metaphor identification in legal Chinese is especially problematic, owing to the fact that many legal words are neologisms and may exhibit less semantic tension at the word level than at the morpheme level. This emerged from the analysis of Chinese data and could not be predicted beforehand. However, for a question of consistency in the methodology of analysing the two sets of data, the identification of linguistic metaphors was performed at the word level in both sets. Future studies are encouraged to consider each of these possibilities and contribute to the development of various under-researched fields of scholarly enquiry such as Chinese legal language, metaphor in Chinese, and, especially, metaphors in Chinese law.

We can now consider the above findings to answer the second question, *whether hybrid languages are in fact ‘system*-*bound’ or if they are ‘value*-*free’*, and, more in general, to relate them with the foci of this special issue—hybridity and Third Space in legal translation.

Hybridity is, as Ashcroft et al. note [5, 118], “one of the most employed and disputed terms in post-colonial theory”. There is no broadly accepted definition for the concept, which, largely speaking, has been used to “refer[] to the creation of new *transcultural* forms within the contact zone produced by colonization” (*ibid*.; emphasis with bold typeface in the original). This is explained by Ashcroft et al. with a horticulture metaphor: hybridity is, thus, the crossbreeding of two species resulting in a third species—a ‘hybrid’. In a sense, the transplantation of the Western concept right into China can be seen as a form of colonization, at least in linguistic terms. Indeed, as I highlighted at the outset of this study, legal transplantation implies translation. *As a result of the legal linguistic colonization, a hybrid language, the Chinese legal language was born*.

The very concept of hybridity draws from a linguistic observation made by philosopher Mikhail Bakhtin (1895–1975), who argued that language “is a concrete heteroglot conception of the world”, given that “*[a] language can represent another language* while still retaining ‘the capacity to sound simultaneously both outside it and within it’” (1994, 293 and 358 in [74, 133]. As Wolf points out, language “is, by its nature, a hybrid construction” [74, 131]. This seems to be especially the case with Chinese legal language, in which the Chinese neologism *quanli* represents Western languages and the Western concept right, a ‘system-bound’ one [12, 23], while still retaining Chinese shades of meaning. As a result, the metaphors in the two languages are different. Additionally, as we have seen, many Chinese legal terms (e.g., *zhuanrang* ‘transfer’, *xingshi* ‘exercise’) are not metaphorical (or they loosely are, as is the case with *qinhai* ‘invade-harm’) in that they are neologisms that display no older basic meaning that contrasts with those they have in the legal context, to which they almost exclusively belong. The metaphor analysis for right in legal Chinese shows that this hybrid jargon has the capacity of simultaneously sounding both within and outside the Chinese legalculture. As has been noted, “Bakhtin (1994, 304–[30]5) showed that frequently even one and the same word belongs simultaneously to two languages or two belief systems that intersect in a hybrid construction” [74, 133]. This is precisely the case of *quanli*, intersecting Western and Chinese belief systems.

It is in light of the above conception of hybridity that Bhabha coined the fortunate term ‘Third Space’, a “multi-dimensional and extremely complex” space [49, 13]. As Ashcroft et al. [5, 61] further explain, Bhabha’s Third Space can be contrasted to Saussure’s view as to how signs acquire meaning, i.e., through their difference from other signs. Similarly, drawing from Saussure, a culture may be identified by its difference from other cultures. In contrast to this, Bhabha’s Third Space is a space of possibility [69, 168], as “a culture’s difference is never simple and static but ambivalent, changing, and always open to further possible interpretation” [5, 61]. Hybridity is, for Bhabha, an empowering phenomenon, as it “helps us to overcome the exoticism of cultural diversity in favour of the recognition of an empowering hybridity” [5, 97]. Thus, culture shall not be conceived of as a monolithic object of study; *rather each and every culture is a space of hybridity* “in which cultural meanings and identities always contain the traces of other meanings and identities” [5, 50]. It is in this vein that Bhabha argues that “claims to inherent originality or purity of cultures are untenable, even before we resort to empirical historical instances that demonstrate their hybridity” [10, 37]. The name ‘Third Space’ is a somewhat vague term coined by Bhabha [10, 36] to precisely capture these phenomena. Through the intervention of the Third Space we realise that any culture is, in a sense, hybrid. As Wolf notes, “[c]ultures are never unitary in themselves, nor simply dualistic as in the relation *self/other* […], rather there is a *Third Space*, which can neither be reduced to the *self* nor the *other*” [74, 135]. This holds true both for Euro English and legal Chinese alike.

Finally, Pozzo [53, 201] proposed that “a hybrid language” such as Euro English “is not connected [to] a given system of values”(*ibid*.). Such hypothesis may potentially imply that all hybrid languages are the same, in that they are all unconnected to any system of value. This hypothesis contrasts with Bhabha’s notion of hybridity and Third Space, for, as we have seen, there can be no language that is free from hybridity, as there is no primordial unity or fixity. The findings of this study seem to confirm Bhabha’s supposition: languages, including hybrid languages, exhibit significant differences. Although both Chinese legal language and Euro English may be regarded as two hybrid languages, and a fundamental Western concept, right, is found in both of them, the fact that its metaphors differ in the two languages show that the legal concept has no primordial unity or fixity, and “‘Third Space’ permits manipulation of the consciousness and unconsciousness of legal discourse” ([70, 2]).

## Conclusion

The conceptualisation of right in the English developed by the European Union institutions and legal Chinese as represented in my data is different, and there is scarce cognitive equivalence between the two, either in terms of conceptual metaphors or in terms of embodied schemas. As a consequence, the embodied simulation of right in the two languaculture is also likely to be different. With the intervention of the Third Space we realise that the very notions of source culture and source language on the one hand, and target culture and target language on the other are simplifications that may readily fit in the translation market, but poorly reflect the complexity and the hybridity of every languaculture. Concepts, including legal concepts, are often manipulated and read anew, showing their primordial unstableness. In this sense, each language variety is a Third Space of its own. While it is somewhat straightforward to conceive of Euro English as a hybrid language, owing to its supranational role in a multilingual context, the Chinese legal language, too, is revealed to be a hybrid and a Third Space.

## Footnotes

1 A conceptual domain is “a body of knowledge within our conceptual system that contains and organises related ideas and experiences” [22, 14]. Conventionally, conceptual domains, such as up, down, time, space, right are written in small caps.

2 The term Mainland China is conventionally used to refer to the territorial entities that directly fall under the Beijing jurisdiction, thus excluding Taiwan, Macau, Hong Kong, and autonomous provinces, where different laws and, in some cases, different legal systems apply.

3 In corpus linguistics, a concordance of a node (aka Key Word in Context or KWIC) is a listing of sentences retrieved by the corpus software, where the node is displayed in the centre and the context in which it appears on its right and left sides.

4 This and more information about the texts contained in the EUR-Lex English 2/2016 corpus can be found at https://www.sketchengine.eu/eurlex-corpus/#toggle-id-2 (last accessed November 24, 2020).

5 Various methods have been adopted to decompose metaphors from complex to primary (see e.g. [40, 34–49; 77]. What we need to note for the purpose of this study is that we cannot (only) compare the metaphor formulae, but we need to gain a more profound understanding of mapping in terms of cross-cultural and cross-lingual comparability by identifying the primary metaphors from which the mapping originates.

6 On the intersubjective accessibility of source domains, see e.g. Brysbaert et al. 2014, p. 904 and Dancyger and Sweetser 2014, both discussed in Winter [73].

7 Separate metaphor research on the universality of rights is being undertaken by the author.

8 It is noted that since ChinLaC is an ongoing project and criminal laws are being uploaded to its database, the logDice of the Chinese collocates discussed here are likely to vary once the upload will be finished. It is believed that this variation will not affect the results, given that criminal laws are a smaller part of the corpus. In a sense, we can regard the conclusions reached in this study as skewed to non-criminal law.

9 See e.g. the #ReframeCovid initiative first proposed by Inés Olza and Paula Sobrino https://sites.google.com/view/reframecovid/home for alternatives to war metaphors for Covid-19.
